# Profiling of Nutraceuticals and Proximates in Peanut Genotypes Differing for Seed Coat Color and Seed Size

**DOI:** 10.3389/fnut.2020.00045

**Published:** 2020-04-15

**Authors:** Spurthi N. Nayak, Viresh Hebbal, Pushpa Bharati, Hajisab L. Nadaf, Gopalkrishna K. Naidu, Ramesh S. Bhat

**Affiliations:** ^1^Department of Biotechnology, University of Agricultural Sciences, Dharwad, India; ^2^Department of Food Science and Nutrition, University of Agricultural Sciences, Dharwad, India; ^3^Department of Genetics and Plant Breeding, University of Agricultural Sciences, Dharwad, India

**Keywords:** groundnut, nutrient profile, polyphenol, antioxidants, proximates, peanut skin color, AhTE markers, marker-trait association

## Abstract

A total of 60 genotypes of peanut comprising 46 genotypes selected from ICRISAT mini core collection and 14 elite cultivars with differing kernel color and size were used to profile the nutritional parameters such as proximates (moisture, fat, ash, crude protein, crude fiber, carbohydrate content) and nutraceuticals (total polyphenol content and total antioxidant activity). The genotypes showed varied kernel color ranging from white to purple. Kernel skin color was quantified using colorimetry, and the color parameters were expressed as CIELAB color parameters. In total, nine morphological traits, six yield related traits, eight nutritional traits and eleven color parameters were observed across 60 genotypes. The sixty genotypes were grouped into ten clusters based on the color strength. Among them, Cluster-III with dark red seeds had the maximum fat content and total polyphenol content (TPC). Cluster-VI with light pink colored seeds had high antioxidant activity (AOA) and Cluster-X with white colored seeds had highest moisture and crude protein content. Color strength (K/S) was found to be positively correlated with TPC. Another color parameter, redness/greenness (a*) was found to be positively correlated with AOA. However, seed size was positively correlated with the crude protein content, but not with any other nutritional traits under study. The population studies based on the genotypic data indicated two distinct groups pertaining to botanical types of peanut. The marker-trait association (MTA) using single marker analysis indicated 75 major MTAs for most of the nutritional traits except for moisture content. The markers associated with nutritional parameters and other important yield related traits can further be utilized for genomics-assisted breeding for nutrient-rich peanuts.

## Introduction

Peanut (*Arachis hypogaea* L.) widely known as groundnut is a cultivated allotetraploid (2n = 4x = 40), particularly valued for its protein content (28%), oil content (50%) and is rich source of calcium, phosphorus, potassium, magnesium, iron, zinc, and boron. The peanuts also contain vitamin E and small amounts of vitamin B complex (1). Due to these properties, it is rightly called as “king of oilseeds” and “poor man's almond” and provides 567 kcal of energy per 100 g of kernels. Peanut is derived from a natural hybridization process between *A*. *duranensis* (AA genome progenitor) and *A*. *ipaensis* (BB genome progenitor) resulted in amphidiploid *A*. *monticola*. Later in the evolutionary process fertile, now cultivated allotetraploid *A*. *hypogaea* was evolved. The genome sequence of cultivated peanut revealed a genome size of 2.7 GB (2, 3). The global production of peanut is estimated to be 47.09 million tons from 27.94 million hectare area with productivity of 1685.6 kg ha^−1^ (4). More than 50% of the peanut produced are used for oil extraction. Rest are either consumed as kernels or processed into various products like peanut butter, milk, cheese analogs, beverages, plumpy nut (a ready to use therapeutic food), and chocolate additives. Peanut is considered as both legume (botanically) and nut (nutritionally). As a plant based protein, it also contains wholesome amount of fiber and other bioactive functional compounds that improve health in humans including heart health, weight loss, gallstone prevention, malnutrition, etc. (5–9). For instance, “Plumpy nuts,” a ready-to-use therapeutic food (RUFT) is being used for the treatment of severe acute malnutrition (SAM) which affect adversely about 20 million children globally, with approximately 8 million cases in India alone. RUTF use is very common in Africa and other developing countries where the incidence of SAM is high. Peanut has several functional components with variety of health benefits like coenzyme Q10 which helps the heart protection under low oxygen areas like high altitude and clogged arteries, rich arginine helps in blood circulation, resveratrol protects against cancer, cognitive disorder and Alzheimer's disease, phytosterols of peanut butter, oil reduces the absorption of cholesterol from the diet, magnesium and dietary fiber reduce the diabetes, reduces LDL cholesterol and helps in weight management [reviewed by (5)]. The foods with different colors including fruits, vegetables, legumes and cereals are found to have functional components that are beneficial for the human health (10). Peanuts have different tinge of kernel colors varying from the white to dark purple with solid or variegated color patterns. The peanut germplasm includes peanut cultivars with varying kernel colors. For selection of appropriate lines that retain the diversity persisting in the global collection becomes essential in utilizing in crop breeding programs. In this regard, the “core collection” (i.e., 10% of the entire germplasm collection) presents a manageable and cost-effective entry point into germplasm collections for identifying candidate genotypes for various traits (11). In peanut, the core collection were further narrowed down to mini core collections and U. S. peanut mini core collection (12), ICRISAT mini core collection (13), and Chinese peanut mini core collection (14). Traits related to abiotic stresses (drought, heat, salinity, low temperature, and P deficiency, calcium induced iron chlorosis), biotic stresses (early leaf spot, late leaf spot, rust, tobacco cut worm, *Aspergillus flavus*, peanut bud necrosis disease, and bacterial wilt) and seed quality (oil, protein, oleic/linoleic ratio, Fe and Zn) have been screened by many researchers in ICRISAT mini core collection (15–17). GWAS for 50 different agronomic traits was carried out in reference set of core collection having 300 genotypes which included the 184 mini core genotypes indicated several markers associated with major agronomic traits (18). The efforts toward elucidating the marker-trait association related to quality or nutritional parameters are limited to some crops like sorghum (19), wheat (20), rice, and barley (21). Evidence of metabotype-phenotype linkage was explained by using parallel metabolite and phenotypic GWAS in traits such as grain color and size in rice and maize kernels (22).

Estimation of proximates like moisture content (MC), fat, crude protein (CP), ash, crude fiber (CF), and carbohydrates (CHO) provide the distribution of major components in any food or processed product. The peanut is rich in nutraceuticals like total polyphenol content (TPC) and total antioxidant activity (AOA). Proximates and nutraceuticals content in the kernel determine the quality of peanut. Peanuts must be dried or cured to guarantee that, the moisture content does not surpass 10.5%, to guarantee quality and to avoid the development of microorganisms. Low moisture percentage of peanut seed prevents it from the susceptibility to the aflatoxin producing fungal pathogens like *Aspergillus flavus* (23). Fatty acid composition in peanut is heart-friendly. Oleic acid (monounsaturated omega-9 fatty acid) is important seed quality parameter and has inverse association with systolic blood pressure. Digestibility of peanut protein is similar to that of animal protein (24). Crude fiber in peanut has little food value but provide the proper help in the intestinal tract for adequate peristaltic action. It contains low glycemic index (GI) and glycemic load (GL) and addition of peanut butter to high glycemic load food can stabilize the blood glucose level of the body. Nutraceuticals of peanut gives health advantages, including the aversion or potential treatment for the diseases [reviewed by (5)].

Peanut pod is made up of external shell (21–29%), kernel (69–73%), germ (2–3.5%), and testa or seed coat (2–3%). Due to papery like structure, seed coat is also called as peanut skin or peanut kernel skin (PKS). Several studies showed that PKS are rich in polyphenol and antioxidant [reviewed by (25)]. The kernel skin is removed through blanching or roasting before the preparation of snack food, groundnut butter and other groundnut-based foods and PKS remained as by-product. High fiber and tannin content of PKS make them usable only for ruminants (and possibly rabbits) but, due to chance of aflatoxin contamination, utilization of PKS as feed remains limited with inclusion rates lower than 10% of the diet as feed for ruminants (26). The reason for the variation in skin color was reported to be due to flavonoid content in cereals, legumes, oilseeds, and several other colored foods (27–29). Due to growing evidence of the versatile health benefits of dietary flavonoids through epidemiological studies (30), the PKS emerges as the potential product to be utilized in diet.

Peanut skin color was found to be strongly associated with total phenolic content and hue angle was proposed to be a biomarker for total polyphenol content and antioxidant capacity when 27 cultivars were screened (31). Similarly total anthocyanin content in the peanut skin was found to be associated with the skin color when 22 selected genotypes from US mini core collection along with 4 Israeli Virginia type cultivars were screened (32). The relation between TPC, flavonoids and AOA was studied with grain color in 481 accessions of rice with white, red and black colored genotypes and correlation studies indicated a negative correlation between a* and antioxidant capacity (33). The grain color was also found to be associated with anthocyanin and Zn content when 156 rice accessions varying in grain color were screened and analyzed through genome-wide association studies (34). Similar findings were revealed from transcriptomics and proteomics studies in colored rice (22). Similarly, GWAS studies have been carried out to dissect the candidate genomic regions for flour color using colorimetric approaches (35).

Different sized peanuts are preferred for different purposes. For instance, peanuts with large seed, low oil but with high oleic acid/linoleic acid (O/L) ratio are preferred for direct consumption, while medium seeded peanuts are preferred by the industries. Studies related to kernel size are limited to inheritance studies (36). A study indicated that kernel size was not significantly correlated with the oil content. Protein content was higher in small seeded compared to the bold seeded peanuts (37). However, in soybean no significant correlation was found between protein and oil content with seed size (38). In peanut the studies on kernel color are limited to the estimation of TPC and flavonoids through biochemical assays and correlation studies thereof. There were limited efforts to identify the genomic regions underlying these traits. In this regard, the molecular markers are considered to be powerful genomic tools to characterize the genetic variation present in the population. The molecular markers can be used in trait mapping and molecular breeding programs [reviewed in (39)]. Low level of genetic variation was observed in peanut. Transposable elements (TE) provide an important source of variation and are highly dynamic in diverse species. Due to the positive correlation of transposable element content with the genome size of the organism, it has been widely known as a genomic parasite and found to be source of variation in plants and animals (40). Use of DNA transposon markers in peanut was proposed by Bhat et al. (41) and presence or absence of *AhMITE1* at a predetermined site in the genome was confirmed using PCR (42). There are two types of transposable elements viz., class I TE (move within genomes via RNA intermediates, using a copy-and-paste mechanism) and class II TE (DNA of a DNA transposon moves by a cut-and-paste mechanism). Class II TEs entail autonomous and non-autonomous elements. Miniature inverted-repeat transposable elements (MITEs) are non-autonomous elements of less than 600 bp in length (43). It contains 10-15 bp terminal inverted repeats and two flanking target site duplications. MITEs are inserted mainly in the gene-rich regions and can affect gene expression (44). In peanut, due to the availability of genome sequence information, several TE markers commonly known as *Arachis hypogaea* transposable element (AhTE) have been identified and are made available in a database at Kazusa Peanut Database (45). AhTE markers have shown greater potential to differentiate the genotypes in groundnut (42, 43, 46). As AhTE markers can be easily screened on agarose gel through electrophoresis, they have been utilized in trait mapping using linkage and association mapping approaches (41–43, 47–51). The candidate genes or genomic regions governing the nutritional traits are presently not available. Hence an effort was made to identify genomic regions that are associated with the nutritional traits including proximates and nutraceuticals like TPC and AOA genotypes that vary in kernel skin color and size.

## Materials and Methods

### Plant Materials and Evaluation of Population

**Figure 1:**
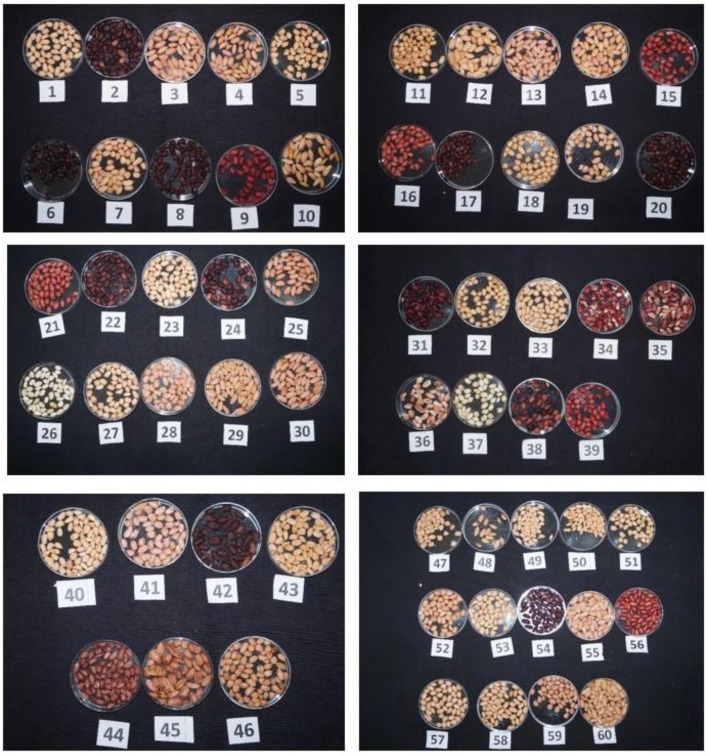
Seeds of sixty peanut genotypes with varying skin color.

A total of 60 genotypes (46 from ICRISAT mini core collection and 14 elite cultivars) were selected based on variation in kernel skin color varying from white to purple color and size (Figure 1). The passport details suggested that they are collected from 25 different countries across the globe (Table 1). Sixty genotypes were evaluated for different morphological and yield related traits by growing them in post-rainy season 2017–18 and rainy season of the year 2018 in a randomized block design with two replications. Each replication consisted of 2 rows of 1.5 m length with a spacing of 30 × 10 cm for the bunch type cultivars (53 genotypes) and 60 × 10 cm for the runners (7 genotypes). Three representative plants were selected randomly from each genotype for recording the phenotyping data on taxonomic and productivity traits. Observations on morphological traits (flower color, stem color, leaf color, leaf shape, growth habit, branching pattern, leaflet length and leaflet width, and plant height) and productivity traits (pod weight per plant, number of pods per plant, shelling percentage, test weight, sound mature kernel weight percentage, and haulm weight) were recorded as per the groundnut descriptor (52).

**Table 1:** List of 60 peanut genotypes with differing skin color and seed size.

**Sl. No**.	**Genotypes**	**Country**	**Sub species**	**Botanical varieties**	**Seed size**	**Color clusters**
1	ICG334	China	*fastigiata*	*vulgaris*	Medium	Cluster-VIII
2	ICG1274	Indonesia	*fastigiata*	*fastigiata*	Small	Cluster-I
3	ICG1668	USA	*hypogaea*	*hypogaea*	Bold	Cluster-VI
4	ICG3053	India	*hypogaea*	*hypogaea*	Medium	Cluster-V
5	ICG3421	India	*fastigiata*	*vulgaris*	Medium	Cluster-VII
6	ICG5475	Kenya	*fastigiata*	*fastigiata*	Medium	Cluster-II
7	ICG5051	USA	*hypogaea*	*hypogaea*	Medium	Cluster-V
8	ICG5221	Argentina	*fastigiata*	*fastigiata*	Bold	Cluster-I
9	ICG5286	Zambia	*hypogaea*	*hypogaea*	Medium	Cluster-II
10	ICG5327	USA	*hypogaea*	*hypogaea*	Small	Cluster-V
11	ICG4670	Sudan	*fastigiata*	*fastigiata*	Small	Cluster-VI
12	ICG5662	China	*hypogaea*	*hypogaea*	Small	Cluster-V
13	ICG1711	Bolivia	*fastigiata*	*fastigiata*	Small	Cluster-III
14	ICG5236	Chile	*fastigiata*	*vulgaris*	Bold	Cluster-III
15	ICG5609	Sri Lanka	*fastigiata*	*fastigiata*	Medium	Cluster-II
16	ICG6201	Cuba	*fastigiata*	*fastigiata*	Medium	Cluster-II
17	ICG6375	Unknown	*fastigiata*	*vulgaris*	Small	Cluster-II
18	ICG6407	Zimbabwe	*fastigiata*	*vulgaris*	Medium	Cluster-VII
19	ICG6646	Unknown	*fastigiata*	*fastigiata*	Small	Cluster-VIII
20	ICG6703	Paraguay	*hypogaea*	*hypogaea*	Small	Cluster-II
21	ICG7181	India	*fastigiata*	*fastigiata*	Small	Cluster-III
22	ICG7190	Brazil	*fastigiata*	*vulgaris*	Small	Cluster-I
23	ICG7963	USA	*hypogaea*	*hypogaea*	Small	Cluster-VIII
24	ICG8106	Peru	*fastigiata*	*fastigiata*	Small	Cluster-I
25	ICG8285	USA	*hypogaea*	*hypogaea*	Medium	Cluster-V
26	ICG9418	Martinique	*fastigiata*	*vulgaris*	Small	Cluster-X
27	ICG9507	Philippines	*fastigiata*	*vulgaris*	Small	Cluster-VII
28	ICG6667	USA	*hypogaea*	*hypogaea*	Medium	Cluster-VI
29	ICG11855	Republic of Korea	*hypogaea*	*hypogaea*	Medium	Cluster-V
30	ICG11862	Republic of Korea	*hypogaea*	*hypogaea*	Small	Cluster-IV
31	ICG12625	Ecuador	*fastigiata*	*aequatoriana*	Medium	Cluster-I
32	ICG12988	India	*fastigiata*	*vulgaris*	Small	Cluster-VIII
33	ICG13982	USA	*hypogaea*	*hypogaea*	Medium	Cluster-VIII
34	ICG14475	Nigeria	*hypogaea*	*hypogaea*	Medium	Cluster-VII
35	ICG12276	Bolivia	*hypogaea*	*hypogaea*	Small	Cluster-IV
36	ICG7243	USA	*hypogaea*	*hypogaea*	Bold	Cluster-IX
37	ICG14710	Cameroon	*fastigiata*	*fastigiata*	Medium	Cluster-X
38	ICG15287	Brazil	*fastigiata*	*vulgaris*	Medium	Cluster-I
39	ICG15309	Brazil	*fastigiata*	*fastigiata*	Small	Cluster-V
40	ICG13723	Niger	*hypogaea*	*hypogaea*	Medium	Cluster-VIII
41	ICG11457	India	*hypogaea*	*hypogaea*	Small	Cluster-VI
42	ICG2381	Brazil	*hypogaea*	*hypogaea*	Bold	Cluster-I
43	ICG5827	USA	*hypogaea*	*hypogaea*	Medium	Cluster-V
44	ICG3992	India	*hypogaea*	*hypogaea*	Medium	Cluster-III
45	ICG8760	Zambia	*hypogaea*	*hypogaea*	Medium	Cluster-VI
46	ICG9037	Cote d'Ivoire	*hypogaea*	*hypogaea*	Small	Cluster-VII
47	ICGV86031	India	*fastigiata*	*vulgaris*	Small	Cluster-VII
48	A30b	India	*fastigiata*	*vulgaris*	Medium	Cluster-VII
49	ICGV06146	India	*fastigiata*	*vulgaris*	Medium	Cluster-VI
50	GPBD5	India	*fastigiata*	*vulgaris*	Small	Cluster-VIII
51	GPBD4	India	*fastigiata*	*vulgaris*	Medium	Cluster-VIII
52	TMV2	India	*fastigiata*	*vulgaris*	Small	Cluster-VII
53	R9227	India	*fastigiata*	*vulgaris*	Small	Cluster-IX
54	DSG1	India	*hypogaea*	*hypogaea*	Medium	Cluster-I
55	ICGV06189	India	*fastigiata*	*fastigiata*	Bold	Cluster-VII
56	Dh40	India	*fastigiata*	*vulgaris*	Small	Cluster-IV
57	Dh245	India	*fastigiata*	*vulgaris*	Small	Cluster-IX
58	Kadari6	India	*fastigiata*	*vulgaris*	Small	Cluster-VII
59	Kadari9	India	*fastigiata*	*vulgaris*	Small	Cluster-VI
60	TGLPS3	India	*hypogaea*	*hypogaea*	Bold	Cluster-V

*The seed size is based on the test weight. The genotypes with <40 g test weight were categorized as “Small,” 40–60 g are categorized as “Medium” and > 60 g were grouped as “Bold” seeded*.

*Color clustering was made as per DMRT analysis, Cluster-I, dark purple colored seeds; Cluster- II, light purple seeds; Cluster-III, dark red; Cluster-IV, light red; Cluster-V, dark pink; Cluster-VI, light pink; Cluster-VII, dark tan; Cluster-VIII, medium tan; Cluster-IX, light tan and Cluster-X, white colored seeds*.

### Estimation of Color Parameters

For identifying the color difference, the color space and coordinates were determined using CIE L*, a*, and b* (CIELAB) values for these genotypes using Spectrophotometer (Fluoro Spectrophotometer-SS5100A, Premier Colorscan) available at AICRP Home Science (CT), UAS, Dharwad. Peanut skin color was quantified in terms of color strength (K/S), reflectance (RFL) and CIELAB (or CIEL*a*b*) color space values (as defined by International Commission on Illumination). CIELAB values include lightness/darkness (L*), redness/greenness (a*), and yellowness/blueness (b*). To identify the color difference, white colored genotype ICG9418 was used as standard and color difference parameters (ΔE, ΔL, Δa, Δb) were derived from CIELAB values. hue angle (h°) an attribute of color perception measure of distinguishing red from green and blue from yellow (expressed in degrees), and chroma (C*) indicating the saturation of the color was determined by using the formulas.

(1) $$\begin{array}{r} \text{hue angle }=\tan^{-1}\left(\frac{{\text{b}}^{*}}{{\text{a}}^{*}}\right) \end{array}$$

(2) $$\begin{array}{r} \text{chroma}=\sqrt{{{\text{a}}^{*}}^{2}+{{\text{b}}^{*}}^{2}.} \end{array}$$

### Estimation of Proximates and Nutraceuticals

The proximates were estimated from the seeds as per the standard protocols given by the Association of Official Analytical Chemists (AOAC), 2005. Seeds were powdered in pestle and mortar and oven dry method was used to determine the moisture content of the sample and stored in desiccator to avoid absorption of moisture from outside. The dry powder was used to estimate the ash content in a muffle furnace. The dry powder was also used to determine the fat content (FC) by using Soxhlet extraction apparatus (SOCS PLUS six place automatic solvent extraction system model SCS 6 AS DLS, Pelican). The defatted moisture free samples were then used to determine the crude protein (CP) by micro-Kjeldahl method (KEL PLUS automatic micro six sample digestion system, Pelican and Kjeldhal automatic nitrogen distillation system (Classic DX VA, Pelican). The crude fiber (CF) was assessed from the defatted samples using alkali and acid digestion followed by ash estimation. The carbohydrate content was determined by differential method. Total polyphenol content (TPC) was determined by using Folin-Ciocalteau reagent (FCR) and catechol (1, 2-dihydroxybenzene) was used as standard. The values were expressed in catechol equivalent (CE) per 100 gram of seeds. Total antioxidant activity (AOA) was determined by using 2, 2-diphenyl-1-picrylhydrazyl (DPPH) reagent and expressed in % DPPH activity or per cent inhibition.

### Genotyping of the Population

The genomic DNA from 60 genotypes was isolated from healthy young leaves using modified CTAB method (53). A total of 100 *Arachis hypogaea* transposable element (AhTE) markers were used to screen these genotypes (Supplementary Table 1). The PCR was carried out in a reaction volume of 10 μl with 50 ng of template DNA, 5 pmol of each primer, 10X of Taq polymerase buffer [500 mM KCl, 100 mM Tris-HCI (pH 8.5)], 0.25 mM of dNTPs and 0.1 U of Taq polymerase. The PCR reaction was carried out in 96 well plates using Mastercycler-PCR (MC Nexus Gradient, Eppendorf AG, Germany) with the temperature profile of initial denaturation of 5 min at 95°C and then 35 cycles at 95°C for 1 min, 53°C for 1 min, and 72°C for 1 min 30 s and 72°C for 8 min for final extension. The amplicons were separated by gel electrophoresis on 1% agarose gels in Bio-Rad gel electrophoresis unit using 1X TAE in buffer tank with electric voltage of 80 V for 1 h.

### Data Analysis

The phenotypic data was analyzed to determine the analysis of variance (ANOVA), frequency distribution and genetic variability for nutritional and yield related traits of sixty peanut genotypes sown during post rainy-2017 and rainy season of 2018 and were calculated at 0.05 alpha value. A null hypothesis was rejected if the F-value > F critical value. Correlation was computed using SPSS 16.0 software. DMRT (Duncan multiple range test) was used for grouping the genotypes based on mean values of color strength. The genotypic data was used to estimate the major allele frequency, heterozygosity, and polymorphic information content (PIC) (54).

The population structure and number of subpopulations were assessed by model-based clustering algorithms using STRUCTURE Version 2.3.4 (55). The number of subpopulations (K) was set from 1 to 10, and at least 500 runs per K were conducted separately with 100,000 generations of “burn-in” and 200,000 Markov chain Monte Carlo (MCMC). The best K value was determined based on delta K analysis (56). Molecular diversity was assessed using NTSYSpc Version 2.0 and DARwin Version 6.0. In order to check if any of the AhTE marker screened on the population is associated with the traits, a marker-trait association study was carried out by using single marker analysis (SMA) using single factor ANOVA. Those significant and major marker-trait associations showing >10% R^2^ were analyzed for their position in the genome and functional annotation using the gene prediction data from diploid progenitor genomes (available at https://peanutbase.org).

## Results

### Variability in Morphological, Yield and Nutritional Traits

In this study, sixty genotypes varying in kernel skin color and size were screened phenotypically for 23 traits including nine morphological, eight nutritional and six yield related traits. Of the 23 traits, six traits (flower color, stem color, leaf color, leaf shape, growth habit, branching pattern) were qualitative in nature and rest 17 traits (leaflet length (cm), leaflet width (cm), plant height (cm), pod weight per plant (g), number of pods per plant, shelling percentage (%), test weight (g), sound mature kernel weight percentage (%), haulm weight (g), and nutritional traits including contents of moisture (%), fat (%), crude protein (%), ash (%), crude fiber (%), carbohydrate (%), and nutraceuticals like TPC (catechol equivalent per 100 g fresh weight) and AOA (% DPPH activity) were quantitative.

The 17 quantitative traits were used to determine the variance value among the sixty genotypes using ANOVA. The results revealed that there were highly significant differences among sixty genotypes for the all the quantitative traits except for haulm weight at 5% probability (Supplementary Table 2). High GCV coupled with PCV was observed for the traits like moisture content (23.13 and 25.52%), total antioxidant activity (20.11 and 20.12%), pod weight per plant (21.9 and 30.84%), and number of pods per plant (20.59 and 34.88%). Low level of GCV and PCV (<10%) was observed for traits like fat, crude protein, crude fiber, leaflet width, shelling percentage, and sound mature kernel weight percentage. Rest of the traits showed moderate GCV and PCV (10–20%). All the nutritional traits except for carbohydrate content (30.32%) studied showed high heritability (>60%). Majority of morphological and yield related parameters showed moderate heritability while leaflet length and width showed high heritability (92.68 and 66.48%). The lowest heritability was observed for haulm weight (11.95%). The highest GA and GAM was observed for TPC (129.35%) and moisture content (43.19%), respectively (Supplementary Table 3).

### Variability in Peanut Skin Color and Size

Among the sixty genotypes, varying degree of kernel skin color was observed that ranged from white to purple. In order to quantitatively determine the peanut skin color, a total of 11 color estimates like K/S, RFL, ΔE, L, a*, b*, ΔL, Δa, Δb, h°, and C* were used. The mean value of the estimates is showed in Supplementary Table 4. Reflectance of the surface of a material can be defined as its effectiveness in reflecting the radiant energy. Color strength is derived from reflectance and it has ability to represent the intensity of color. It can differentiate high intensity color with faded colors having low intensity. The genotypes were found to be highly differing with respect to intensity of skin color. For grouping the genotypes with similar color, we used color strength as reference parameter using DMRT analysis. A total of 10 clusters differing significantly for color strength at 5% probability were formed. The proximates and nutraceuticals were assessed according to the groups differing with color strength. The Cluster-I consisted of majority of dark purple colored seeds, Cluster-II with light purple seeds, Cluster-III dark red, Cluster-IV light red, Cluster-V dark pink, Cluster-VI light pink, Cluster-VII dark tan, Cluster-VIII medium tan, Cluster-IX light tan, and Cluster-X with white colored seeds.

The color strength decreased from Cluster-I (dark purple) to Cluster-X (white). Cluster-I having dark purple colored seeds showed the lowest crude proteins and moisture content and high range of fat content and crude fiber. Cluster-III with dark red colored seeds showed highest fat and TPC content and lowest crude fiber. Cluster- IV with light red colored seeds showed highest moisture, fat, ash, crude fiber contents. Cluster- VI with light pink colored seeds showed highest AOA and lowest fat content. Cluster-X with white colored genotypes showed the lowest fat content, TPC and AOA and highest crude protein content and moisture content. Other clusters showed intermediate range of nutritional parameters (Table 2).

**Table 2:** Clusters based on color strength of the kernel skin color using Duncan's multiple range test (DMRT).

**Traits**	**MC**	**Fat**	**CP**	**Ash**	**CF**	**CHO**	**TPC**	**AOA**
Cluster-I	3.85^cd^	45.04^ab^	25.98^e^	2.53^d^	11.19^ab^	11.28^a^	359.76^e^	68.94^i^
Cluster-II	3.89^cd^	44.73^ab^	27.58^cd^	2.45^f^	11.02^abc^	10.35^a^	380.70^c^	71.90^h^
Cluster-III	4.74^abc^	45.31^a^	27.63^bcd^	2.50^e^	10.63^c^	9.19^a^	429.77^a^	86.27^b^
Cluster-IV	5.18^a^	45.51^a^	26.07^e^	3.11^a^	11.42^a^	8.70^a^	392.85^b^	76.03^g^
Cluster-V	4.52^abcd^	43.84^bc^	28.03^bc^	2.39^g^	10.59^c^	10.62^a^	344.36^f^	78.41^e^
Cluster-VI	4.91^ab^	42.62^c^	28.17^b^	2.50^e^	11.09^abc^	10.71^a^	385.62^bc^	86.68^a^
Cluster-VII	3.76^d^	44.71^ab^	27.28^d^	2.57^c^	10.72^bc^	10.96^a^	335.16^f^	82.24^c^
Cluster-VIII	4.19^bcd^	43.74^bc^	27.25^d^	2.30^h^	10.96^abc^	11.55^a^	299.82^g^	81.53^d^
Cluster-IX	4.19^bcd^	44.97^ab^	27.93^bc^	2.74^b^	11.48^a^	8.69^a^	369.62^d^	77.36^f^
Cluster-X	5.28^a^	42.56^c^	29.58^a^	2.39^g^	10.96^abc^	9.22^a^	269.99^h^	25.64^j^

*Within columns, mean values are followed by the same letters (like a, b, c, etc.) are not significantly different (P < 0.05) according to Duncan's multiple range test (DMRT)*.

*MC, Moisture content; Fat, Fat content; CP, Crude protein content; Ash, Ash content; CF, Crude fiber content; CHO, Carbohydrate content; TPC, Total polyphenol content; AOA, Total antioxidant activity; K/S: Color strength*.

Assessment of kernel size was based on the test weight of the seeds. Genotypes with test weight less than 40 g were considered as small seeded, those with the range of 40–50 g were considered as medium sized and with more than 50 g of test weight were recorded as bold seeded genotypes (Table 1).

### Phenotypic Correlation

The degree of relationship between kernel size along with nutritional traits, color parameters and yield related traits were computed using Pearson's correlation test. Coefficients of correlation were computed to assess the magnitude and direction of relation between the traits. Among the 420 possible correlations from 29 quantitative traits (kernel size, 8 nutritional, 11 color parameters, 3 morphological and 6 yield related traits), a total of 113 trait pairs were found to be significant at 5% probability (Supplementary Table 5). Kernel size showed positive correlation with crude protein and test weight. Carbohydrate content showed negative correlation with moisture, fat, ash, crude fiber, and TPC. Fat content showed negative correlation with crude protein content. Crude protein content showed negative correlation with K/S, ΔE and h° while it showed positive correlation with RFL, L*, ΔL, b*, Δb, and chroma value. Fat content showed positive correlation with ΔE and h° while it showed negative correlation with b* value. TPC was found to be positively correlated with K/S and ΔE while it showed negative correlation with RFL, ΔL, L*, and b* value. AOA showed positive correlation with a* and Δa. Number of pods per plant showed negative correlation with plant height and positive correlation with haulm weight and pod weight per plant. Sound mature kernel weight percentage is positively correlated with pod weight per plant and test weight.

### Molecular Diversity and Population Studies

Sixty genotypes of groundnut were genotyped with 100 AhTE markers. A total of 59 markers were found to be monomorphic across 60 genotypes while 41 AhTE markers showed polymorphism. The polymorphic markers were utilized to study the molecular diversity, population structure and marker-trait associations. To understand the ability of markers to differentiate the genotypes, major allele frequency, heterozygosity, and PIC were computed. Major allele frequency ranged from 0.51 (AhTE1542) to 0.98 (AhTE1438, AhTE1587) with a mean of 0.84. Heterozygosity of the markers ranged from 0.03 (AhTE1587) to 0.47 (AhTE0446) with a mean of 0.23. The PIC value ranged from 0.03 (AhTE1438 and AhTE1587) to 0.37 (AhTE0205, AhTE0474, and AhTE1542) with mean PIC value of 0.19 (Supplementary Table 6).

**Figure 2:**
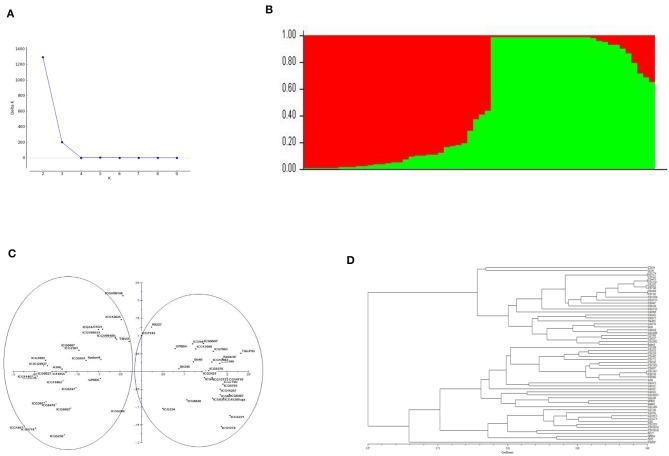
**(A)** Population structure of the sixty genotypes of peanut. **(B)** Bar plot representing the number of subpopulations. **(C)** Principal co-ordinate analysis (PCoA). **(D)** Dendrogram representing the grouping of genotypes based on AhTE markers genotyping data.

Population structure analysis showed two major groups based on delta K value when genotyping data from 41 polymorphic markers were used in analysis. The principal coordinate analysis (PCoA) and the dendrogram also showed the presence of two major groups among the genotypes (Figure 2). These two groups clearly showed to be consisting of genotypes with different botanical varieties. The genotypes exhibited significant phenotypic differences with respect to morphological, nutritional, colorimetric and yield related parameters but with moderate genetic diversity explained by 41 polymorphic markers among them.

### Marker-Trait Association Analysis

The genotypic data from 41 polymorphic AhTE markers across 60 genotypes along with phenotypic data on 28 traits including 8 nutritional, 11 color parameters, 3 morphological and 6 yield related traits were subjected for single marker analysis (SMA) to study the marker-trait associations (MTAs). A total of 36 markers (110 MTAs) showed the significant association at 5% probability level. In total 75 MTAs (contributed by 30 markers) showed major marker-trait associations with PVE value more than 10% (Table 3) and about 35 MTAs showed the PVE less than 10%. An MTA, AhTE1542-Haulm weight, had the highest PVE value (27.68 %). Maximum number of marker-trait associations (43 MTAs) were detected for 9 morphological and yield related traits (leaflet length, leaflet width, plant height, number of pods per plant, pod weight per plant, shelling percentage, test weight, sound mature kernel weight percentage, and haulm weight), followed by nutritional (24 MTAs) for 7 traits (fat, crude protein, ash, crude fiber, carbohydrate, TPC, and total AOA) and 8 MTAs were detected for 6 color parameters (color strength, reflectance, L*, a*, Δa, hue angle). Out of 75 major MTAs, two MTAs (AhTE0474-AOA and AhTE0362-leaflet length) were significant at 0.1% P-value and six MTAs (AhTE1542-haulm weight, AhTE0296-plant height and leaflet length, AhTE0189-TPC and leaflet length) were significant at 0.05% P-value. Two highly significant markers (AhTE0474 and AhTE0189) were strongly associated with total AOA and TPC, respectively. The marker-trait associations for some of the important nutritional traits (fat content, crude protein content, ash content, crude fiber content, TPC, and AOA) is represented in Manhattan plot constructed by plotting position on chromosome against negative logarithm to the base 10 of observed p value of all the polymorphic AhTE markers (Supplementary Figure 1).

**Table 3:** Association of molecular markers for nutritional, color, morphological, and yield related traits using single marker analysis in sixty peanut genotypes.

**Sl. No**.	**Chromosome**	**Marker**	**Traits**	***P*-value**	**F value**	**PVE**
1	A01	AhTE0233	PH	0.0256^*^	3.92	12.69
2			AOA	0.0158^*^	4.48	14.23
3	A02	AhTE0465	LL	0.0077^**^	7.64	12.01
4			LW	0.0078^**^	7.63	11.98
5			SP	0.0011^**^	11.83	17.44
6		AhTE1098	SP	0.0037^**^	9.16	13.84
7	A03	AhTE0205	HW	0.0156^*^	6.27	11.14
8			SP	0.0036^**^	9.33	15.72
9			PH	0.0099^**^	7.19	12.57
10			AOA	0.0162^*^	6.19	11.01
11		AhTE0442	TPC	0.0430^*^	3.34	11.39
12		AhTE0474	AOA	0.0008^***^	12.49	18.25
13	A04	AhTE1253	PWPP	0.0056^**^	8.33	13.57
14			TW	0.0082^**^	7.55	12.48
15	A05	AhTE1271	PH	0.0461^*^	3.25	10.57
16		AhTE1277	Fat	0.0417^*^	3.36	10.50
17			CHO	0.0146^*^	4.55	13.78
18			LL	0.0025^**^	6.63	18.87
19			LW	0.0295^*^	3.74	11.62
20			PH	0.0166^*^	4.40	13.38
21			SP	0.0066^**^	5.47	16.12
22	A06	AhTE1337	CHO	0.0059^**^	8.18	12.55
23		AhTE1363	Ash	0.0047^**^	8.73	14.87
24			SMKW	0.0216^*^	5.62	10.10
25		AhTE1379	LL	0.0146^*^	6.34	10.35
26	A08	AhTE1437	PH	0.0042^**^	6.06	18.32
27	A10	AhTE1542	CP	0.0447^*^	3.29	11.06
28			AOA	0.0278^*^	3.83	12.63
29			LL	0.0422^*^	3.36	11.26
30			PH	0.0097^**^	5.06	16.05
31			NPPP	0.0120^*^	4.81	15.36
32			SP	0.0020^**^	6.96	20.81
33			HW	0.0002^***^	10.14	27.68
34		AhTE1564	HW	0.0369^*^	3.50	11.30
35	B01	AhTE1581	Ash	0.0095^**^	7.20	11.39
36			CHO	0.0098^**^	7.14	11.30
37			K/S	0.0117^*^	6.77	10.79
38			RFL	0.0017^**^	10.77	16.13
39			HW	0.0046^**^	8.71	13.46
40	B02	AhTE0296	SP	0.0036^**^	9.24	14.62
41			PH	0.0003^***^	14.58	21.25
42			LL	0.0003^***^	14.58	21.25
43	B04	AhTE0189	a^*^	0.0195^*^	5.86	11.29
44			AOA	0.0073^**^	7.89	14.65
45			TPC	0.0003^***^	14.97	24.56
46			LL	0.0001^***^	16.60	22.85
47		AhTE1744	HW	0.0137^*^	4.65	14.92
48		AhTE1761	Fat	0.0095^**^	5.08	15.84
49			CP	0.0088^**^	5.16	16.06
50		AhTE1777	Δa	0.0088^**^	7.37	11.82
51			h°	0.0068^**^	7.92	12.59
52		AhTE1780	SP	0.0361^*^	3.53	11.57
53	B05	AhTE0446	AOA	0.0350^*^	3.57	11.67
54			PH	0.0080^**^	8.08	23.05
55			SP	0.0425^*^	3.35	11.04
56	B06	AhTE1872	CHO	0.0069^**^	5.48	17.40
57		AhTE1907	PH	0.0105^*^	6.70	10.93
58			SP	0.0035^**^	9.30	14.02
59			TW	0.0068^**^	7.89	12.15
60			HW	0.0037^**^	9.19	13.88
61		AhTE1916	RFL	0.0073^**^	7.78	12.59
62	B07	AhTE0143	PH	0.0002^***^	16.09	22.96
63			LL	0.0090^**^	12.35	18.61
64			AOA	0.0038^**^	9.15	14.49
65			TPC	0.0144^*^	6.38	10.58
66	B08	AhTE2000	Fat	0.0162^*^	4.47	14.67
67			CP	0.0280^*^	3.83	12.85
68			CF	0.0133^*^	4.67	15.3
69			SP	0.0124^*^	4.78	15.52
70		AhTE2011	K/S	0.0116^*^	4.87	16.04
71			L^*^	0.0078^**^	5.34	17.32
72			TW	0.0011^**^	7.85	23.53
73	B09	AhTE0362	TPC	0.0046^**^	8.75	14.17
74			LL	0.0008^***^	12.57	19.17
75			SP	0.0175^*^	6.01	10.18

*** *Significant level at 0.5%*,

** Significant level at 1%, and

* *Significant level at 5%*.

PVE, Phenotypic variance; Fat, Fat content; CP, Crude protein; Ash, Ash content; CF, Crude fiber content; CHO, Carbohydrate content; TPC, Total polyphenol content; AOA, Total antioxidant activity; K/S, Color strength; RFL, Reflectance; L*, Lightness value; a*, Redness-greenness of the color; Δa, Redness or greenness difference between sample and standard colors; h°, Hue angle; LL, Leaflet length; LW, Leaflet width; PH, Plant height; PWPP, Pod weight per plant; NPPP, Number of pods per plant; SP, Shelling percentage; TW, Test weight; SMKW, Sound mature kernel weight percentage; HW, Haulm weight.

Among the 30 AhTE markers showing significant major MTA, a few were associated with multiple traits. For example, AhTE1542 showed the strong association with two nutritional traits like crude protein (11.06%) and AOA (12.63%) and five yield related traits like leaflet length (11.26%), plant height (16.05%), number of pods per plant (15.36%), shelling percentage (20.81%), and haulm weight (27.68%). AhTE1277 showed association with two nutritional traits like fat content (10.5%) and carbohydrate content (13.78%) and four yield related traits like leaflet length (18.87%), leaflet width (11.62%), plant height (13.38%), and shelling percentage (16.12%). AhTE1581 showed association for 5 traits including color parameters like color strength (10.79%), reflectance (16.13%), nutritional traits like ash content (11.39%), carbohydrate content (11.30%) and yield related trait like haulm weight (13.46%). Five markers, AhTE0143 (plant height, leaflet length, TPC, and AOA), AhTE0189 (a*, AOA, TPC, and leaflet length), AhTE0205 (haulm weight, shelling percentage, plant height, and AOA), AhTE1907 (plant height, shelling percentage, test weight, and haulm weight), and AhTE2000 (fat, crude protein, crude fiber and shelling percentage) showed the association for 4 traits each. AhTE2000 showed maximum number of association with 3 nutritional traits like fat content (14.67%), crude protein content (12.85%) and crude fiber content (15.30%). Among the quality traits AhTE0189-TPC, showed the highest PVE (24.56%). Maximum number of marker-trait associations was observed for markers on chromosome number B04 (5 AhTE markers with 10 MTAs) (Table 3).

### Prediction of Candidate Genes using Associated Markers

The 30 AhTE markers that showed major MTAs were used to predict the probable candidate genes. Except for A09, B03, and B10, the associated markers were distributed on all other chromosomes. Out of 30 AhTE markers, 3 contains *AhMITE1* insertion site in intron, 6 in upstream, 4 in downstream, 1 UTR, 1 exon and 15 in intergenic regions (Table 2, Supplementary Table 7). For instance, the AhTE1542 that showed insertion at the exonic region of *Aradu.S8151* [codes for U3 small nucleolar ribonucleoprotein (MPP10-like protein)] on chromosome A10 showed significant association with seven traits including crude protein, total AOA, leaflet length, plant height, number of pods per plant, shelling percentage, and haulm weight. Similarly, the marker AhTE0442 showed significance association with TPC. *AhMITE1* insertion was at intronic region of the gene *Aradu.UKZ71* on chromosome A03 that codes for catalytic activity or oxidoreductase activity. AhTE1437 showed significant association with plant height. *AhMITE1* insertion was found at intronic region of the gene *Aradu.Z3TSR* on chromosome A06. The gene codes for serine type carboxypeptidase activity. The marker AhTE0205 showed the significant association with haulm weight, shelling percentage, plant height, and AOA. The *AhMITE1* insertion was found at the intronic region of gene *Aradu.SV32V* on chromosome A03, that codes for uncharacterized protein LOC100811541 isoform X2 (*Glycine max*).

Among the 30 markers associated with nutritional, color parameters, morphological, and yield related traits, majority (15) were found to be reside on intergenic regions. The marker AhTE1761 corresponded to intergenic region (between *Araip.A758G* and Araip. *7QQ9G*) on chromosome B04 showed significant association with fat and crude protein content. The candidate genes showed function of stress induced protein. Similarly, AhTE1363 with intergenic position (*Aradu.ULX7G* and *Aradu.SDQ6B*) on chromosome A06 showed significant association with ash content and sound mature kernel weight percentage. The candidate genes showed the function zinc ion transmembrane transporter activity. The intergenic marker AhTE2000 located between genes *Araip.TVQ3P* and *Araip.H5SDY* on chromosome B08, was associated with fat content, crude protein, crude fiber and shelling percentage. The candidate genes showed the function of structural constituent of cell wall protein. Another intergenic marker AhTE0189 located between genes *Aradu.WFR54* and *Aradu.05MV1* on chromosome B04 showed significant associated with color parameter a* value, AOA, TPC, and leaflet length. The candidate genes showed the function of receptor-like kinase activity (Supplementary Table 7).

## Discussion

Genetic improvement of peanut for quality and yield traits is essential for combating the malnutrition and hunger in the developing countries. Major focus of the peanut breeding programs hovers around increasing the yield potential of the crop to increase the productivity under biotic and abiotic stresses. Advances in genomics technologies in peanut through next generation sequencing, have deciphered tremendous genomic resources that can be utilized in genomics-assisted breeding. In this regard, efforts were made to identify molecular markers or genomic regions associated with traits such as late leaf spot and rust (57–61), aflatoxin contamination (62), drought (63), yield related traits (50), and some quality traits like protein content, oil content, and oil quality (64–66). Another important trait that needs attention in peanut is its skin color- more commonly called as peanut kernel skin (PKS) color. The association of skin color with various nutritional parameters is well established in various vegetables and fruits (30). In food crops, the relationship between flavonoids and antioxidant activity was studied with grain color in colored rice (33, 34). However, in peanut the studies are limited to studying the relation between PKS with nutraceuticals like total polyphenol content and antioxidant capacity (31, 32). In this context this, present study reports the profiling of nutritional parameters in sixty genotypes of peanut that differ in skin color and size.

Colorimetric method was used for quantification of skin color that had estimated 11 color parameters. For color estimation, there are many color models like munsell color space, RGB/CMYK, YIQ/YUV/YCbCr, HIS/HSV/HSL, Hunter Lab color space, and CIEXYZ/CIEL*U*V*/CIEL*a*b*. Among these models CIE model and Hunter Lab color space are commonly used for evaluation of color difference while other have their application area in computer graphics, image analysis, and processing, etc. (67, 68). Both the color space are based on opponent-colors theory (69). In the present study CIELAB values were derived by using the colorimetric approach to quantify the peanut skin color. Similar approach was used to study the skin color in 27 peanut cultivars (31), 481 colored rice accessions (33). Similarly hunter scale was used for quantification of color estimation of skin color of 22 US peanut mini core collection and 4 Israeli cultivars (32), 17 colored chickpea lines (70), and 33 cool season legumes (71). These color parameters were also considered as the biomarkers for estimating the TPC and AOA in peanut skin (31). Hence color strength was used for clustering the 60 genotypes used in the study by DMRT analysis and 10 distinct groups were made. Cluster-I consisted of dark purple while cluster-X with white colored genotypes. Cluster-X with white colored genotypes showed the lowest fat content, TPC, and AOA indicating light skin colored genotypes were poor in the nutraceuticals while dark colored seeds are rich in nutraceuticals compared to white seeded genotypes. Similar observations were recorded in peanut (31, 32), rice (33), chickpea (70). In this study light pink colored seeds showed highest antioxidant activity rather than expected dark purple colored seeds, which may be due to colorless flavonoid that exhibit strong antioxidant activity. Similar finding i.e. high TPC was observed in light pink colored peanut kernel skin (32). Cluster-X with white skin colored genotypes showed highest protein content and lowest fat content, further the study also showed the negative correlation between fat content and protein content. This clearly suggests that lighter skin color is an indicator of high protein and low fat content. The flavonoids were found to have inhibitory action on protein production in eukaryotes (72, 73). In the present study peanut skin was used to assess the correlation with nutritional parameters in the whole kernel (including skin), that provides better understanding of the nutritional status of the peanut seeds. There are some reports where nutraceuticals were measured in peanut skin devoid of kernel (32). Presently, there are no published reports to correlate proximates with skin color in peanut; hence this study will provide the prime information on proximate profiling. As expected, white colored genotypes showed lowest reflectance value and highest moisture content. The moisture content was lowest in dark tan (Cluster-VII) followed by dark and light purple colored (Cluster-I and II) seeds which contain lower moisture content maybe due to their high light absorbance nature. Generally, colored/darker grains have higher nutritional value compared to their light counterparts. For instance, the dark colored rice were nutrient rich compared white rice (74). In this study also colored seeds showed higher nutritional value compared to white and light colored seeds except crude protein content. The AOA in the present study was comparatively higher in colored seeds than the white ones; this kind of observation was also evident in rice (33). The TPC content was found to be highest in dark red (Cluster-III) colored seeds followed by light red (Cluster-IV) and lowest in white seeds (Cluster-X). But, the findings from other peanut skin studies found that light pink colored skins followed by red had larger amount of TPC content (32). The crude fiber content was maximum in light red (Cluster-IV) and light tan (Cluster-IX) and minimum in dark red and dark pink seeds (Cluster III and V).

The inheritance studies of peanut skin color carried out way back by Higgins (75) shows that the flesh-colored seed testa is dominant over white color with bigenic difference, whereas the red testa color is dominant to flesh-colored testa with monogenic difference. It also showed the necessity of flesh pigment for the expression of red color. These results were further confirmed on different white colored peanuts by Hammons (76). However, Norden *et al*. (77) revealed the presence of single dominant gene that was epistemic to the previously described genes for testa color in peanut. A contradictory observation where, white testa color was found to be dominant in one of the genotypes (78). Further, three different alleles were identified for the red testa color and among them; R1 allele was to be dominant over the other two (r2 and r3) recessive alleles (79). In another study, the purple and purple striped- testa in peanut showed monogenic and bigenic inheritance respectively (80). The observations made on the inheritance studies in peanut showed debatable results, indicating the complex nature of seed coat color. Since then, there are limited efforts to elucidate the mechanisms involved in seed coat color. The present study provides some insight into seed coat color and its association with AhTE markers like, AhTE1581-color strength, AhTE0189-reddness-greenness of the color, AhTE2011-ligthness-darkness of the color, AhTE1777-hue angle of the kernel skin color. Further investigations are necessary to understand the molecular mechanisms underlying testa color determination using holistic genomics and metabolomics approaches.

Generally, Virginia peanut cultivars are bold seeded, majority of Valencia are medium sized and Spanish types are small seeded genotypes. In the present study based on test weight genotypes were classified into small, medium, and bold seeded types. The kernel size had no effect on any of the nutritional parameters except for crude protein according to the correlation studies. A positive correlation of kernel size with protein content was observed which was contrary to the previous observation in peanut by Prathiba and Reddy (37). However, no significant correlations between seed size with protein content was observed in soybean (38). Hence the relation between kernel size and protein content is debatable and needs further investigation.

Correlation studies showed the negative association of fat content and protein which was in accordance with the findings by Dwivedi et al. (81). Number of pods per plant showed negative correlation with plant height and positive correlation with pod weight per plant. As the plant height increases, the peg penetrating the soil to make pod decreases. Hence the number of pods per plant as well as pod weight per plant decreases (82). In the present study, number of pods per plant and pod weight per plant showed high GCV coupled with PCV which was also in accordance with the findings of Hake et al. (50).

The studies on seed color and their relation with other nutrients in peanut and many other crops was restricted to the biochemical analysis and lacked the molecular approach to identify probable candidate/ genomic regions. Hence for molecular characterization, AhTE markers that show higher polymorphism were utilized in this study with PIC value ranging from 0.03 to 0.37. The heterozygosity ranged from 0.03 to 0.47, with mean 0.23 across the genotypes. This may be due to the biallelic nature of AhTE markers and also the type of population which consist of diverse genotypes. This finding was in accordance with previous peanut studies (50, 51). About 41 out of 100 AhTE markers screened across 60 genotypes were found to be polymorphic. The genotypic data was used to study the structure of the population. The study revealed that there were two subpopulations that were grouped based on the botanical types. Though variability was found with respect to phenotype, genotypically the population showed less variation. Population structure was analyzed in different populations in peanut (like mini core collections, reference collections, mutant populations) and found that the grouping was made as per the botanical varieties of *Arachis hypogaea*, which include *hypogaea, hirsuta, fastigiata, vulgaris* etc. (18, 50, 83).

Most of these traits are quantitative in nature and are influenced by number of minor genes. The single marker analysis showed about 110 MTAs with 75 major and 35 minor MTAs. Out of which, 43 MTAs were detected for 9 morphological and yield related traits (leaflet length, leaflet width, plant height, number of pods per plant, pod weight per plant, shelling percentage, test weight, sound mature kernel weight percentage, and haulm weight). AhTE0465 (leaflet length, leaflet width, and shelling percentage), AhTE1253 (pod weight per plant and test weight), and AhTE1907 (plant height, shelling percentage, test weight and haulm weight) showed association with only morphological and yield related traits. The marker AhTE0205 on chromosome number A03 showed association with plant height. In another study, a SNP marker with location A03-26481539 was found to be associated with plant height in peanut using GWAS approach (84).

Few MTAs were also observed for color parameters like AhTE1581 (K/S and RFL), AhTE0189 (a*), AhTE1777 (hue angle and Δa), AhTE2011 (L*), and localized on chromosomes B01, B04, and B08. A genome wide association study in peanut also indicated a SNP with location B03-22076736 position had significant association with seed coat color (44). The occurrence of associated markers on B genome might indicate the genome dominance for color parameter that needs to be further investigated.

Out of 75 major MTAs, 24 were related to nutritional traits. Markers associated with AOA include AhTE0233, AhTE0205, AhTE0474, AhTE1542, AhTE0189, AhTE0446, AhTE0143, and highest PVE (18.25%) was explained for AhTE0474 which is localized on chromosome A03. Markers associated with TPC include AhTE0442, AhTE0189, AhTE0362, AhTE0143 and highest PVE (24.56%) was explained for AhTE0189. AhTE0189 and AhTE0143 were fund to be associated with both the nutraceuticals i.e., TPC and AOA. Trait mapping in sorghum through GWAS found candidate genes that are associated with TPC (19). Except for moisture content, markers associated with all the proximates were identified in this study. For example, AhTE2000 marker localized at chromosome B08 was associated with fat content, crude protein and crude fiber. Similarly, AhTE1761 on chromosome B04 was found be associated with fat content and crude protein content. In peanut, association studies on quality parameters using molecular markers were limited to oil content, protein, Fe and Zn content and no published records are available for proximates and nutraceuticals. Previous reports on genome-wide association on reference set of peanut consisting of 300 genotypes showed MTAs for quality traits like, oil (25 MTAs), oleic acid (2 MTAs) protein (11 MTAs), oleic/linoleic acid (22 MTAs), and zinc (1 MTA) (18). Also, highly significant 38 MTAs for protein and oil related traits were observed (50).

The associated markers were checked for their location on the peanut genome from PeanutBase. The chromosome B04 had five markers that were found to be associated with a*, AOA, TPC, leaflet length, haulm weight, fat, crude protein, Δa, hue angle, and shelling percentage. Chromosomes A03, A06, and B06 had 3 markers each that were found to be associated with 5 traits (haulm weight, shelling percentage, plant height, AOA, and TPC), 4 traits (carbohydrate, ash, sound mature kernel weight percentage, and leaflet length) and 6 traits (carbohydrate, plant height, shelling percentage, test weight, haulm weight, reflectance), respectively. Further gene annotation for the candidate genes of some of the marker like AhTE0442, AhTE0205, and AhTE0474 on chromosome number A03 showed functions related to oxidoreductase activity. Several reports in peanut showed markers associated with disease resistance were also localized on A03 that was evident from QTL mapping and sequencing (59, 60, 85). The occurrence of genes encoding for antioxidant activity at A03 might provide disease resistance in peanut. However, large number of markers needs to be screened on a larger population to identify the candidate genes/markers using genome-wide association analysis to validate these findings. Further, the associated markers after validation and identified genotypes with favorable alleles can be utilized in molecular breeding for nutritionally rich peanuts.

## Conclusions

In the current scenario, improving the peanut crop with quality parameters is equally important along with increasing yield and pest resistance. In order to combat the malnutrition and hunger, we need to work toward “more nutrition per bite.” This study provides relation of peanut skin color with various nutritional parameters (proximates, nutraceuticals), morphological and yield related traits. The markers associated with important traits after validation and genotypes with favorable alleles can be utilized in genomics-assisted peanut improvement.

## Data Availability Statement

All datasets generated for this study are included in the article/Supplementary Material.

## Author Contributions

SN and HN conceived the presented idea. VH performed the experiment. GN provided the seed material. SN, PB, and HN designed the experiments. RB provided the molecular markers. PB facilitated lab for nutrition estimation and helped in conducting nutritional experiments. SN and VH analyzed the data, and wrote the manuscript. SN supervised the project and arranged for funding.

### Conflict of Interest

The authors declare that the research was conducted in the absence of any commercial or financial relationships that could be construed as a potential conflict of interest.
