# Supplementary material for: Profiling of Nutraceuticals and Proximates in Peanut Genotypes Differing for Seed Coat Color and Seed Size
Source: Front Nutr. 2020 Apr 15;7:45. doi: 10.3389/fnut.2020.00045 (PMC7174653; doi:10.3389/fnut.2020.00045)
Supplement: Supplementary file 1 [file Data_Sheet_1.docx]

**AhTE1761 AhTE1761**


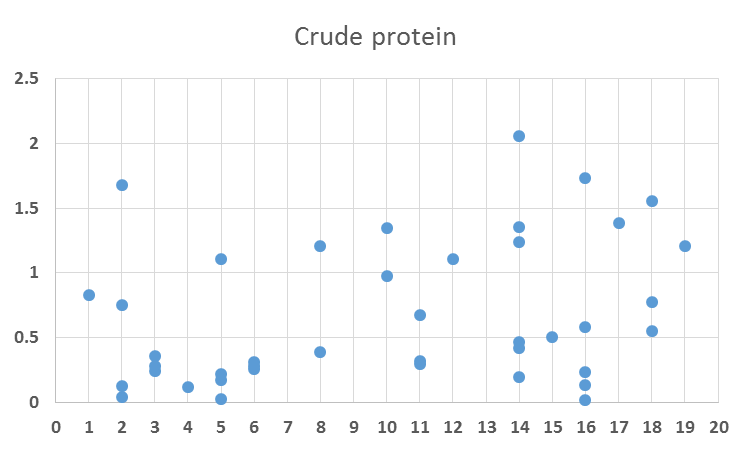

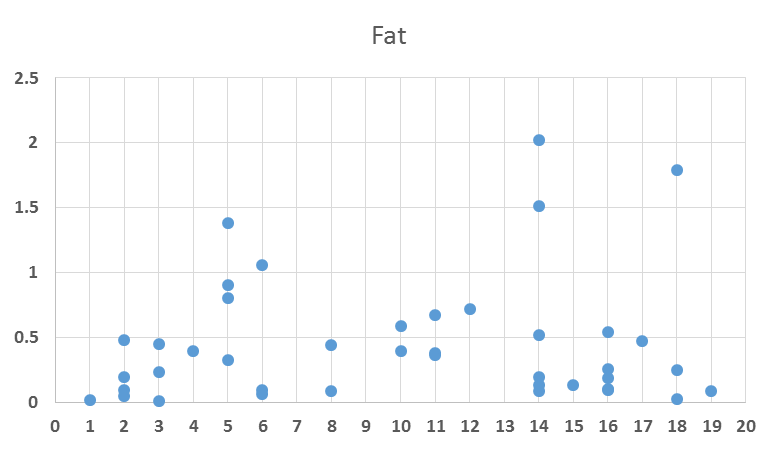


**–Log_10_(p)**

**–Log_10_(p)**

**Position Position**

**a) Fat content b) Crude protein content**


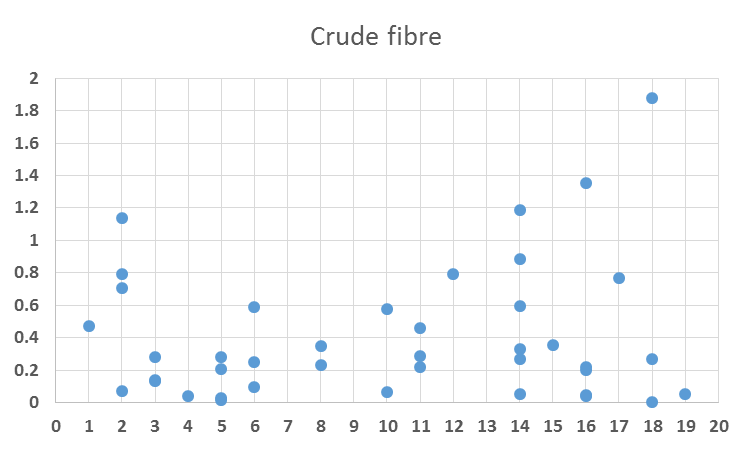

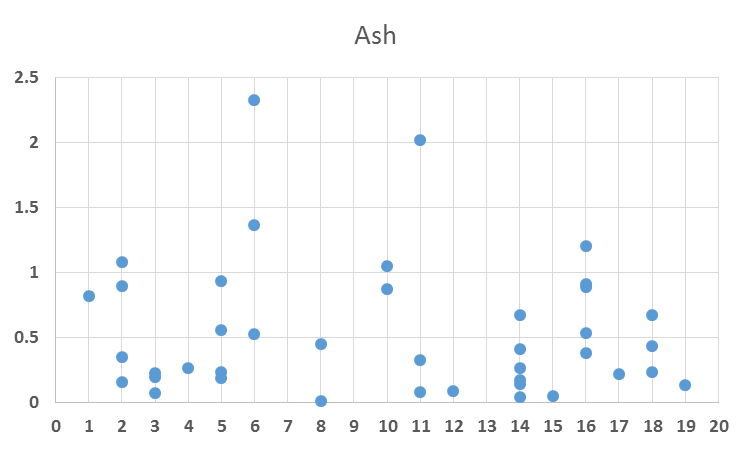
  **AhTE1363 AhTE2000**

**–Log_10_(p)**

**–Log_10_(p)**

**Position Position**

**c) Ash content d) Crude fibre content**


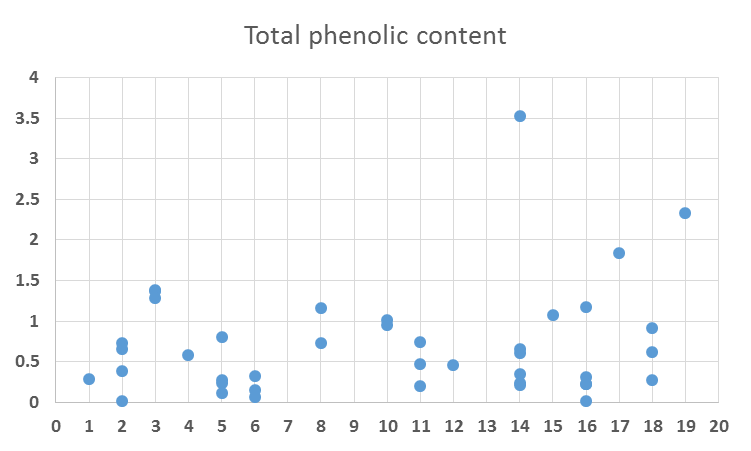

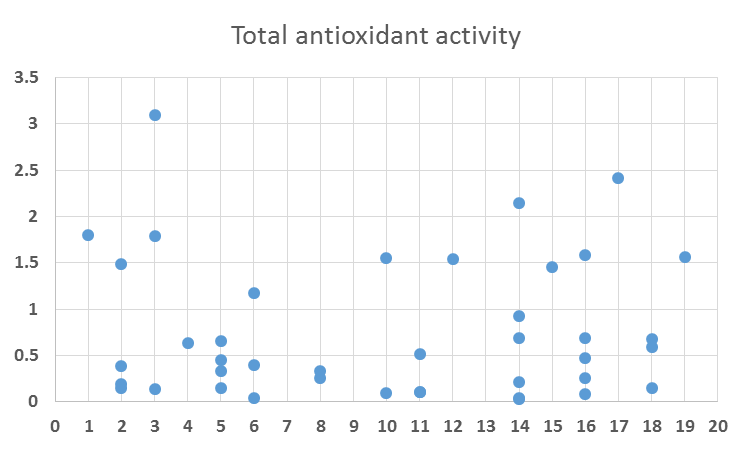
  **AhTE0189 AhTE0474**

**–Log_10_(p)**

**–Log_10_(p)**

**Position Position**

**e) Total polyphenolic content e) Total antioxidant activity**

**Supplementary Figure 1: Manhattan plot showing association of markers with nutritional traits like fat content, crude protein, ash, crude fibre, total polyphenolic content and total antioxidant activity**
